# Effect of timing of protein and carbohydrate intake after resistance exercise on nitrogen balance in trained and untrained young men

**DOI:** 10.1186/1880-6805-33-24

**Published:** 2014-08-06

**Authors:** Hiroyasu Mori

**Affiliations:** 1 Faculty of Health Science, Department of Nutrition Management, Hyogo University, 2301 Arazaike, 675-0195 Hiraoka-cho, Kakogawa City, Hyogo, Japan

**Keywords:** Trained, Untrained, Young men, Nutrient intake timing, Protein, Carbohydrate, Nitrogen balance

## Abstract

**Background:**

Resistance exercise alters the post-exercise response of anabolic and catabolic hormones. A previous study indicated that the turnover of muscle protein in trained individuals is reduced due to alterations in endocrine factors caused by resistance training, and that muscle protein accumulation varies between trained and untrained individuals due to differences in the timing of protein and carbohydrate intake. We investigated the effect of the timing of protein and carbohydrate intake after resistance exercise on nitrogen balance in trained and untrained young men.

**Methods:**

Subjects were 10 trained healthy men (mean age, 23 ± 4 years; height, 173.8 ± 3.1 cm; weight, 72.3 ± 4.3 kg) and 10 untrained healthy men (mean age, 23 ± 1 years; height, 171.8 ± 5.0 cm; weight, 64.5 ± 5.0 kg). All subjects performed four sets of 8 to 10 repetitions of a resistance exercise (comprising bench press, shoulder press, triceps pushdown, leg extension, leg press, leg curl, lat pulldown, rowing, and biceps curl) at 80% one-repetition maximum. After each resistance exercise session, subjects were randomly divided into two groups with respect to intake of protein (0.3 g/kg body weight) and carbohydrate (0.8 g/kg body weight) immediately after (P0) or 6 h (P6) after the session. All subjects were on an experimental diet that met their individual total energy requirement. We assessed whole-body protein metabolism by measuring nitrogen balance at P0 and P6 on the last 3 days of exercise training.

**Results:**

The nitrogen balance was significantly lower in the trained men than in the untrained men at both P0 (*P* <0.05) and P6 (*P* <0.01). The nitrogen balance in trained men was significantly higher at P0 than at P6 (*P* <0.01), whereas that in the untrained men was not significantly different between the two periods.

**Conclusion:**

The timing of protein and carbohydrate intake after resistance exercise influences nitrogen balance differently in trained and untrained young men.

## Background

During exercise, skeletal muscle metabolizes proteins in response to motor stimuli. When protein synthesis exceeds protein breakdown, the positive nitrogen balance promotes muscle growth [1,2]. In particular, high-intensity resistance exercise increases the synthesis of muscle protein for up to 24 h after exercise [1-4]. Because muscle protein synthesis peaks immediately after exercise and reduces over time, intake of dietary protein immediately after resistance exercise is important for muscle protein accumulation [5-7]. Furthermore, the simultaneous intake of protein and carbohydrate facilitates this accumulation because carbohydrate intake inhibits muscle protein breakdown after resistance exercise [8-12]. Regular resistance exercise has been reported to reduce the turnover between synthesis and degradation of muscle protein in healthy individuals in their 20s. For example, muscle protein synthesis in trained young individuals was found to continue for only 36 h after resistance exercise [3] compared with 48 h in untrained young individuals [4]. In addition, investigation of endocrine factors that influence metabolic balance in muscle protein revealed that trained individuals had a low level of testosterone and cortisol secretion after resistance exercise [13]. Resistance exercise also altered the post-exercise response of anabolic and catabolic hormones [14]. These results indicate that the turnover of muscle protein in trained individuals is reduced due to alterations in endocrine factors caused by resistance training, and that muscle protein accumulation varies between trained and untrained individuals due to differences in the timing of protein and carbohydrate intake. In this study, we investigated the effects of protein and carbohydrate intake timing after resistance exercise training by calculating nitrogen balance in trained and untrained young individuals. Proper timing of protein and carbohydrate intake after resistance exercise depends on the amount of each in everyday meals. In particular, total energy intake (TEI) and total protein intake greatly affect nitrogen balance. To test our hypothesis, we monitored the dietary intake of subjects while properly balancing TEI and total energy expenditure (TEE).

## Methods

**Figure 1:**
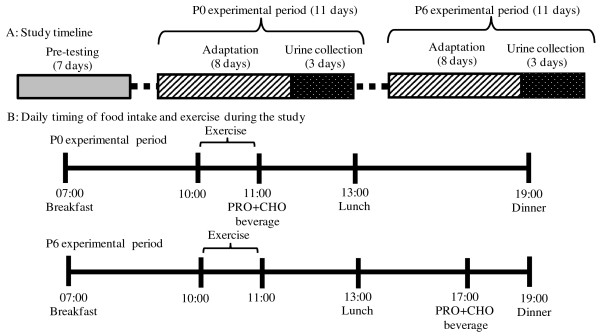
**Experimental schedule.** Subjects were intake of protein (0.3 g/kg body weight) and carbohydrate (0.8 g/kg body weight) immediately after (P0 experimental period) or 6 h (P6 experimental period) after resistance exercise session. PRO, Protein; CHO, carbohydrate.

Of the 20 healthy adult men aged 20 to 29 years enrolled in this study, those who regularly performed resistance exercise were assigned to the trained group (n = 10; mean age, 23 ± 4 years; height, 173.8 ± 3.1 cm; weight, 72.3 ± 4.3 kg) and those who did not were assigned to the untrained group (n = 10; mean age, 23 ± 1 years; height, 171.8 ± 5.0 cm; weight, 64.5 ± 5.0 kg), respectively. The subjects in the trained group were three weightlifters and seven bodybuilders who had trained for 6.2 ± 2.8 years and performed resistance training two to five times a week. The subjects in the untrained group participated in recreational sports such as baseball or soccer two to three times a week, but had no prior experience in resistance exercise. The study protocol was approved by the Ethics Committee of Hyogo University and was conducted in compliance with the Declaration of Helsinki. Subjects were informed verbally and in writing of the content and potential risks of this study in advance and gave their written consent to participate. The following two experimental conditions were used in this randomized crossover study. In the P0 experimental period, subjects consumed protein and carbohydrate supplements 5 min after resistance exercise, and in the P6 experimental period, subjects consumed the same supplements 6 h after exercise. A washout period >7 days was applied before each experimental period. During each 11-day experimental period, the first 8 days were defined as an adaptation period for muscle to adapt to the energy and nutrients from the experimental food and supplements prepared by the examiner. During the next 3-day period (day 9 to day 11), 24-h urine samples were collected. The two experimental schedules are shown in Figure 1. Because at least 7 days of adaptation and 3 days of urine collection are needed to calculate nitrogen balance [6], the resistance exercise schedule in the P0 and P6 experiments lasted for 11 days: 8 days of adaptation (two cycles of resistance exercise for 3 days and rest for 1 day) and 3 days of urine collection. Prior to each experimental period, body composition and one-repetition maximum (1RM) were measured, and questionnaires on daily activity were completed. A body composition analyzer (InBody430; Biospace, Tokyo, Japan) was used to measure weight in 0.1 kg increments, and bioelectrical impedance analysis was performed to calculate percent body fat (%) and lean body mass (LBM). Body composition was measured during the 3-day period (days 9 to 11) when 24-h urine samples were collected. One-repetition maximum was measured in bench press, shoulder press, triceps pushdown, leg extension, leg press, leg curl, lat pulldown, rowing, and biceps curl. Subjects wore a triaxial accelerometer (Active Style Pro HJA-350IT; Omron Healthcare Co., Ltd., Tokyo, Japan) during the 7-day pre-testing periods, except when sleeping or taking a bath, and daily TEE prior to exercise was determined for each subject. In daily experimental sessions, subjects performed the following resistance exercises: 4 sets of 8 to 10 repetitions of resistance exercise consisting of leg press, leg extension, and leg curl on experimental days 1, 5, and 9; bench press, shoulder press, and triceps pushdown on experimental days 2, 6, and 10; and lat pulldown, biceps curl, and rowing on experimental days 3, 7, and 11. All exercises were performed at 80% RM, and each set was followed by a 2-min break. Before the experimental session each day, subjects used a cycle ergometer (Aerobike 800; Combi Wellness Corporation, Tokyo, Japan) at 100 W for 10 min to warm-up. Each exercise session was scheduled to take place between 10:00 and 11:00. The subjects were instructed not to participate in any other sports activities during the experimental period. The protein supplement used in this study was a whey protein powder (Big Whey; Bulk Sports, Miyagi, Japan) containing 78.4 g protein, 6.0 g lipid, and 8.4 g carbohydrate per 100 g product, as used in our previous studies [15,16]. The composition of essential amino acids was 4,400 mg valine, 8,800 mg leucine, 4,600 mg isoleucine, 7,500 mg lysine, 1,600 mg methionine, 2,600 mg phenylalanine, 4,500 mg threonine, and 1,300 mg tryptophan [15,16]. The amount of protein per meal was defined as follows:

$$\mathrm{Protein}\;\mathrm{intake}\;\left(g\right)\;\mathrm{per}\;\mathrm{meal}\;=\;\mathrm{weight}\;\left(\mathrm{kg}\right)\;\times \;0.3$$

This study also used a carbohydrate supplement (Power Gel; PowerSports, Kamakura, Japan) containing 0.0 g protein, 0.0 g lipid, and 73.2 g carbohydrate (dextrin as the main component) per 100 g of the gelatinized form of the product [15,16]. The amount of carbohydrate per meal was defined as follows:

$$\begin{array}{l} \mathrm{Carbohydrate}\;\mathrm{intake}\;\left(g\right)\;\mathrm{per}\;\mathrm{meal}\\ \;=\;\mathrm{weight}\;\left(\mathrm{kg}\right)\;\times \;0.8 \end{array}$$

The protein and carbohydrate supplements were solubilized in 200 mL mineral water for oral consumption 5 min after resistance exercise (at around 11:00) during the P0 experimental period or 6 h after resistance exercise (at around 17:00) during the P6 experimental period. Based on the findings of previous studies [5-9,11-13], we considered that 0.3 g/kg body weight protein and 0.8 g/kg body weight carbohydrate are required to maximize insulin secretion and muscle protein synthesis [15,16]. In addition to the supplements described above, we prepared a specific diet for subjects during each experimental period and instructed them to refrain from consuming other foods. For fluid replacement, subjects were instructed to refrain from consuming high-calorie drinks, but were allowed to consume 0-calorie drinks such as mineral water and green tea. During the experimental periods, breakfast, lunch, and dinner were consumed at around 7:00, 13:00, and 19:00, respectively. A registered dietitian determined target calorie and nutrient intake and target food groups in line with TEE estimated from the daily activity questionnaire and in accordance with the Dietary Reference Intakes for Japanese (2010) [17] and the Nutritional and Dietary Guidelines for Athletes [18]. Furthermore, daily total protein intake from the experimental diet and protein supplement was set at 1.5 g/kg body weight, and calorie and nutrient intake was calculated as described in our previous study [15]. Table 1 shows target calorie and nutrient intake in an experimental diet. Each meal was planned by the dietitian based on individualized target calorie, nutrient, and food group requirements in a way that minimized excess or deficiency. Leftovers from meals were examined carefully to determine the actual calorie and nutrient intake during experimental periods. In the dietary questionnaire, the weighing method and 24-h recall method were used to record the content of meals consumed during the 7-day pre-testing periods and the P0 and P6 experimental periods. TEI, total protein intake, and protein intake per body weight (kg) (hereafter, protein/body weight) were calculated by a registered dietitian using Excel Eiyo-kun software (ver. 6.0; Kenpakusha, Tokyo, Japan). A special container for urine (U-Container; Sumitomo Bakelite Co., Ltd., Tokyo, Japan) was used to perform 24-h urine collection during the 3-day period from experimental day 9 to 11. Resistance exercise was not performed on experimental day 8. The urine samples for experimental day 9 comprised urine collected on day 9, except for the first morning sample, plus the first urine sample on day 10. The same procedure was repeated for experimental days 10 and 11. On day 12, the final urine sample was collected in the morning, and the urinary urea nitrogen levels in the collected samples were analyzed using the urease/glutamate dehydrogenase/ultraviolet method (N-Assay BUN-L Nittobo; Nittobo Medical Co., Ltd., Tokyo, Japan). Nitrogen balance was calculated as follows:

**Table 1:** Nutritional information of the experimental diet

	**Trained group (n = 10)**	**Untrained group (n = 10)**
TEI (kcal)	3,170 ± 125	2,750 ± 222
Protein (g)	108.5 ± 6.4	96.6 ± 7.4
Protein (g/kg body weight)	1.5	1.5
Calcium (mg)	650	650
Iron (mg)	7.5	7.5
Vitamin A (μgRE)	750	750
Vitamin B_1_ (mg)	1.4	1.4
Vitamin B_2_ (mg)	1.6	1.6
Vitamin C (mg)	100	100

Values are means ± SD.

TEI, total energy intake.

$$\begin{array}{ll} \;\mathrm{Nitrogen}\;\mathrm{balance}\;\left(g\right)= & \mathrm{nitrogen}\;\mathrm{intake}\;-\;\mathrm{nitrogen}\\ & \mathrm{excretion}\;(\mathrm{urine}\;\mathrm{urea}\;\mathrm{nitrogen}\;\mathrm{level}\\ & +\mathrm{fecal}\;\mathrm{nitrogen}\;\mathrm{excretion}\\ & +\mathrm{miscellaneous}\;\mathrm{nitrogen}\;\mathrm{excretion})\text{.} \end{array}$$

The equation assumes fecal nitrogen excretion to be 5 mg/kg body weight and miscellaneous nitrogen excretion to be 2 g/kg body weight. To determine nitrogen intake, daily total protein intake calculated from the experimental diet and supplements consumed for the day was divided by the nitrogen coefficient 6.25. The difference between the levels of nitrogen intake and nitrogen excretion is the nitrogen balance. The values of nitrogen balance obtained on experimental days 9, 10, and 11 during each experimental period were averaged to obtain mean nitrogen balance, and mean nitrogen balance per body weight (kg) and per LBM (kg) was also calculated. Statistical analysis was performed using IBM SPSS Statistics 21 software (SPSS Inc., Tokyo, Japan). The unpaired t test was used to analyze pre-testing physical characteristics in both the trained and untrained groups and the paired t test was used to analyze nitrogen balance between P0 and P6 in each group. Nitrogen balance was also analyzed by two-way analysis of variance to compare the experimental conditions (P0 and P6) and groups (trained and untrained). When interactions were found, Tukey’s test was used to adjust confidence intervals to investigate simple main effects. Significance was set at *P* <0.05.

## Results

Pre-testing physical characteristics are shown in Table 2. In the trained group, body weight, BMI, LBM, physical activity level (PAL), TEE, 1RM leg press, 1RM bench press, and 1RM lat pulldown were significantly higher than in the untrained group (weight, BMI, 1RM leg press, and 1RM bench press, *P* <0.01; LBM, PAL, TEE, and 1RM lat pulldown, *P* <0.001), while body fat was significantly lower (*P* <0.05). The amounts of protein and carbohydrate in supplement drinks were 21.7 ± 1.3 and 57. 9 ± 3.4 g in the trained group and 19.3 ± 1.5 and 51.5 ± 4.0 g in the untrained group, respectively. The corresponding amounts in lunch were 32.1 ± 2.1 and 154.5 ± 6.1 g in the trained group and 29.1 ± 2.1 and 134.1 ± 10.8 g in the untrained group. Table 3 shows the comparison of the two groups in relation to nitrogen balance and nutrient intake during the experiments. During the P0 experimental period, the trained group had significantly higher urine urea nitrogen, nitrogen excretion, and nitrogen intake (all *P* <0.01) than the untrained group, but a significantly lower nitrogen balance (*P* <0.05), nitrogen balance/weight (*P* <0.01), and nitrogen balance/LBM (*P* <0.01). Likewise, during the P6 experimental period, the trained group had a significantly higher urine urea nitrogen (*P* <0.001), nitrogen excretion (*P* <0.001), nitrogen intake (*P* <0.01) than the untrained group, but a significantly lower nitrogen balance (*P* <0.01), nitrogen balance/weight (*P* <0.01), and nitrogen balance/LBM (*P* <0.001). No difference was observed in total protein intake per kilogram body weight between the two groups during the experimental periods. The comparison of P0 and P6 in the trained group showed that urine urea nitrogen and nitrogen excretion were significantly lower for P0 (both *P* <0.01), whereas nitrogen balance, nitrogen balance/weight, and nitrogen balance/LBM were significantly higher (all *P* <0.01). However, no differences were observed between P0 and P6 in the untrained group. Under both experimental conditions, TEI and total protein intake were significantly higher in the trained group (TEI, *P* <0.001; total protein intake, *P* <0.05) than in the untrained group, even though total protein intake per kilogram body weight did not differ between the groups.

**Table 2:** Physical characteristics of 20 trained and untrained young men

	**Trained group (n = 10)**	**Untrained group (n = 10)**
Age (years)	23 ± 4	23 ± 1
Height (m)	173.8 ± 3.1	171.8 ± 5.0
Body weight (kg)	72.3 ± 4.3**	64.4 ± 5.0
BMI (kg/m^2^)	23.9 ± 1.2**	21.8 ± 1.3
Body fat (%)	12.7 ± 2.7*	15.6 ± 2.5
LBM (kg)	63.1 ± 3.8***	54.3 ± 3.3
PAL	1.8 ± 0.1***	1.7 ± 0.1
TEE (kcal)	3,204 ± 165***	2,692 ± 257
1RM leg press (kg)	163.5 ± 16.2**	140.0 ± 15.1
1RM bench press (g)	88.1 ± 5.8**	75.6 ± 9.6
1RM lat pull down (kg)	74.5 ± 7.6***	58.5 ± 5.2

Values are means ± SD.

Differences from Untrained group,*P <0.05, **P <0.01, ***P <0.001(paired Student’s t test). BMI, body mass index; LBM, lean body mass; PAL, physical activity level; TEE, total energy expenditure; 1RM, one repetition maximum.

**Table 3:** Nitrogen balance and nutrient intake in the young men immediately and 6 h after exercise

	**Trained group (n = 10)**		**Untrained group (n = 10)**		***P *value**^**1)**^		
	**P0**	**P6**	**P0**	**P6**	**Group experiment (Trained group *vs. *(P0 *vs. *P6) Untrained group)**		**Group *vs. *experiment**
Urinary volume (mL/24 h)	1,617 ± 130	1,618 ± 97	1,501 ± 138	1,536 ± 125	0.060	0.459	0.494
Urinary urea nitrogen (g/24 h)	10.4 ± 0.8** †	10.9 ± 0.7***	9.0 ± 0.8	9.1 ± 0.9	0.001	0.001	0.005
Nitrogen excretion (g/24 h)	16.0 ± 1.0** †	16.6 ± 0.8***	14.2 ± 1.1	14.3 ± 1.1	0.001	0.001	0.005
Nitrogen intake (g/24 h)	17.1 ± 1.1**	17.1 ± 1.1**	15.5 ± 1.1	15.5 ± 1.1	0.002	0.624	0.961
Nitrogen balance (g/24 h)	1.1 ± 0.3* †	0.5 ± 0.4**	1.3 ± 0.1	1.2 ± 0.2	0.001	0.001	0.014
Nitrogen balance/body weight (mg/24 h)	15.3 ± 4.0** †	7.4 ± 5.5***	20.4 ± 2.3	19.0 ± 3.1	0.001	0.001	0.009
Nitrogen balance/LBM (mg/ 24 h)	17.4 ± 4.3** †	8.4 ± 0.2**	24.2 ± 2.8	22.5 ± 3.5	0.001	0.001	0.011
TEI (kcal)	3,187 ± 122**	3,109 ± 125**	2,734 ± 220	2,729 ± 230	0.001	0.541	0.652
Protein intake (g)	107.0 ± 7.0**	106.9 ± 6.7	96.9 ± 6.9	96.8 ± 6.8	0.012	0.621	0.412
Protein intake (g/kg body weight)	15.0 ± 0.1	15.0 ± 0.1	15.0 ± 0.1	15.0 ± 0.1	0.543	0.612	0.546

Values are means ± SD.

^**1)**^By two-way ANOVA with repeated measures analysis of variance with post hoc Tukey’s tests.

Differences from Untrained group, *P <0.05, **P <0.01, ***P <0.001.

Differences from P6, †*P* <0.01.

LBM, lean body mass; P0, Protein and carbohydrate intake immediately after resistance exercise session; P6, Protein and carbohydrate intake 6 h after resistance exercise session; TEI, total energy intake.

## Discussion

This study aimed to elucidate how the timing of protein and carbohydrate intake after resistance exercise affects nitrogen balance differently in trained and untrained men with a different number of years of resistance training experience. Because the meals for each subject were determined by a dietitian, we were able to accurately assess the effect of protein and carbohydrate intake timing after resistance exercise under conditions appropriately balancing TEI and total protein intake. The results revealed a significantly lower nitrogen balance in the trained group than in the untrained group under both conditions (P0 and P6). The findings also showed that, compared with the untrained group, the trained group had a lower level of muscle protein accumulation regardless of the timing of protein and carbohydrate intake. According to Cadore et al. [13], the levels of secreted anabolic hormones associated with muscle protein growth, including systemic testosterone and sex hormone-binding globulin, were lower after resistance exercise in long-term trained men than in untrained men. Moreover, Moore et al. [14] reported that 12 weeks of training downregulated the synthesis and breakdown of muscle protein after resistance exercise. In addition, regular resistance training was reported to lower muscle protein turnover rates in the trained men, suggesting that the speed of recycling amino acids produced by the catabolism of muscle protein during resistance training is reduced. This is in keeping with the present findings showing that muscle protein accumulation in the untrained group was higher than in the trained group after resistance exercise under both the P0 and P6 conditions.

Nitrogen balance in the trained group was significantly higher at P0 than at P6, suggesting that muscle protein in these men was effectively accumulated in response to protein and carbohydrate supplementation if given immediately after exercise. Tang et al. [19] reported that 8 weeks of unilateral leg training reduced the time required to reach peak muscle protein synthesis after resistance exercise. Furthermore, according to Burd et al. [1], the synthesis of muscle protein occurs within a shorter window in trained men than in untrained men. In the present study, we observed no difference in nitrogen balance between the P0 and P6 conditions in the untrained group. In a study conducted by Rasmussen et al. [20] in which untrained men consumed essential amino acids and glucose either 1 or 3 h after resistance exercise, it was found that although muscle protein synthesis was increased under both conditions, no difference was observed in muscle protein accumulation. Furthermore, Tang et al. [19] showed that untrained skeletal muscles took more than 4 h to reach peak protein synthesis after exercise, but that protein synthesis was maintained for several hours. One of the characteristics of whey protein, which was used in this study, is that it is absorbed quickly by the digestive system and is released into the blood approximately 1 to 2 h after intake [21]. As mentioned above, no difference in nitrogen balance was observed between the P0 and P6 conditions in the untrained group, which may have been due, in part, to the inability of the untrained men, whose post-exercise protein synthesis peaks later, to accumulate sufficient protein from the whey protein, which is quickly digested and absorbed immediately after exercise. However, because the untrained group could maintain muscle protein synthesis for many hours, they were able to increase their accumulation of muscle protein even when the timing of protein and carbohydrate intake was late, as in the P6 experimental condition. Although this study showed that the timing of whey protein and carbohydrate intake after exercise did not affect the accumulation of muscle protein in the untrained men, we did not investigate changes in the secretion of endocrine hormones known to affect the anabolism and catabolism of muscle protein after exercise. Further study is therefore needed to investigate in more detail how the timing of protein and carbohydrate intake affects muscle protein accumulation in trained and untrained men. In the present experiments, we estimated target calorie and nutrient intake and set total protein intake to 1.5 g/kg body weight in both groups. However, while this value was equivalent to 1.7 ± 0.1 g/kg LBM in the trained group, it corresponded to 1.8 ± 0.1 g/kg LBM in the untrained group, representing a significant difference between the two groups (*P* < 0.05). This means that compared with the trained group, nitrogen balance in the untrained group was high under both conditions, suggesting that the higher total protein intake per LBM in the untrained group affected the results. Because of the differences in body fat and LBM between the two groups, the evaluation of nitrogen balance in both groups was effective when total protein intake for the experimental period was estimated using LBM, rather than body weight. It may be necessary to review the method for calculating total protein intake when determining target calorie and nutrient intake for trained and untrained men with very different body compositions. Our previous study investigated how differences in the timing of protein and carbohydrate intake after 7 weeks of resistance exercise training affected skeletal muscle accumulation in trained young men and revealed no increase in skeletal muscle mass when whey protein and carbohydrate were consumed immediately after resistance exercise [15]. In the present study, the trained group showed less muscle protein accumulation than in the untrained group even though the former showed a higher effect of taking protein and carbohydrate immediately after resistance exercise, indicating that their skeletal muscle mass hardly increased compared with that observed in the 7-week resistance training period in our previous study. In a separate study of trained men conducted by Hoffman et al., skeletal muscle mass did not increase with protein supplementation before or after 10 weeks of resistance training [22]. However, in a study of untrained men, Bird et al. reported an increase in skeletal muscle mass after taking essential amino acids and carbohydrate over 12 weeks of resistance training [8]. Taking these findings into account, we assume that the anabolic function of insulin secretion after protein and carbohydrate intake was lower in trained men with low muscle protein turnover rates than in untrained men. To verify this hypothesis, further study is needed to carefully investigate the insulin secretion response to protein and carbohydrate intake in trained and untrained individuals. In addition, by taking both our present and previous findings into consideration, we plan to investigate the differential effects of the timing and duration of protein and carbohydrate intake after resistance exercise on the accumulation of skeletal muscle mass. This study has two limitations. First, the effect of protein and carbohydrate intake timing was not investigated in either group when not participating in resistance exercise. Consequently, it is not clear whether the present findings reflect the effect of protein and carbohydrate supplements consumed by trained and untrained men or the timing of supplement intake after resistance exercise. We plan to develop additional experimental methods to carefully investigate the effects of protein and carbohydrate intake timing after resistance exercise. The second limitation was the use of nitrogen balance to evaluate muscle protein metabolism. Many studies have investigated muscle protein metabolism in response to resistance exercise by the indicator amino acid oxidation method or muscle biopsy [2-5,7]. Although invasive, both methods enable detailed evaluation of transient muscle protein metabolism or amino acid kinetics compared with evaluating nitrogen balance and are especially appropriate for investigating the timing of nutrient intake after exercise, as in this study. In contrast, evaluating nitrogen balance has not been widely used to investigate the timing of nutrient intake after exercise even though it is the main approach for determining the equilibrium and balance of energy intake or the daily total protein requirement. Yet, Jordan et al. used nitrogen balance to investigate the timing of protein intake after exercise because its effect depends on daily TEI and total protein intake [6]. This suggests that in addition to the indicator amino acid oxidation method and muscle biopsy, nitrogen balance can be used to accurately evaluate the effect of nutrient intake timing, including total energy and protein intake, after resistance exercise. We will continue to investigate the effect of protein and carbohydrate intake timing using different methods.

## Conclusions

The aim of this study was to investigate how differences in the timing of protein and carbohydrate intake after resistance exercise affect nitrogen balance in healthy trained men and healthy untrained men. The findings indicate that the post-exercise accumulation of muscle protein in the trained men who had continuously performed resistance exercise was low compared with that of the untrained men. Furthermore, when protein and carbohydrate were consumed immediately after resistance exercise, the effect of protein intake on muscle protein accumulation was high in the trained men, but no effect was observed in the untrained men.

## Competing interests

The author declares that he has no competing interests.

## Author contributions

HM conceived the study, collected data, performed statistical analysis, and wrote the manuscript.

## Author information

Hiroyasu Mori: Faculty of Health Science, Department of Nutrition Management, Hyogo University, Hyogo, Japan, Hiroyasu Mori: h-mori@hyogo-dai.ac.jp.

## References

[B1] Burd NA Tang JE Moore DR Phillips SM Exercise training and protein metabolism: influences of contraction, protein intake, and sex-based differences J Appl Physiol 2009 106 1692 1701 10.1152/japplphysiol.91351.2008 19036897

[B2] Chesley A MacDougall JD Tarnopolsky MA Atkinson SA Smith K Changes in human muscle protein synthesis after resistance exercise J Appl Physiol 1992 73 1383 1388 1280254 10.1152/jappl.1992.73.4.1383

[B3] MacDougall JD Gibala MJ Tarnopolsky MA MacDonald JR Interisano SA Yarasheski KE The time course for elevated muscle protein synthesis following heavy resistance exercise Can J Appl Physiol 1995 20 480 486 10.1139/h95-038 8563679

[B4] Phillips SM Tipton KD Aarsland A Wolf SE Wolfe RR Mixed muscle protein synthesis and breakdown after resistance exercise in humans Am J Physiol 1997 273 E99 E107 9252485 10.1152/ajpendo.1997.273.1.E99

[B5] Borsheim E Tipton KD Wolf SE Wolfe RR Essential amino acids and muscle protein recovery from resistance exercise Am J Physiol Endocrinol Metab 2002 283 48 57 10.1152/ajpendo.00466.2001 12217881

[B6] Jordan LY Melanson EL Melby CL Hickey MS Miller BF Nitrogen balance in older individuals in energy balance depends on timing of protein intake J Gerontol A Biol Sci Med Sci 2010 65 1068 1076 20622139 10.1093/gerona/glq123 PMC3031452

[B7] Roy BD Tarnopolsky MA MacDougall JD Fowles J Yarasheski KE Effect of glucose supplement timing on protein metabolism after resistance training J Appl Physiol 1997 82 1882 1888 10.1063/1.365993 9173954

[B8] Bird SP Tarpenning KM Marino FE Liquid carbohydrate/essential amino acid ingestion during a short-term bout of resistance exercise suppresses myofibrillar protein degradation Metabolism 2006 55 570 577 10.1016/j.metabol.2005.11.011 16631431

[B9] Borsheim E Cree MG Tipton KD Elliott TA Aarsland A Wolfe RR Effect of carbohydrate intake on net muscle protein synthesis during recovery from resistance exercise J Appl Physiol 2004 96 674 678 10.1152/japplphysiol.00333.2003 14594866

[B10] Hamada K Matsumoto K Minehira K Doi T Okamura K Shimizu S Effect of glucose on ureagenesis during exercise in amino acid-infused dogs Metabolism 1998 47 1303 1307 10.1016/S0026-0495(98)90296-7 9826204

[B11] Miller SL Tipton KD Chinkes DL Wolf SE Wolfe RR Independent and combined effects of amino acids and glucose after resistance exercise Med Sci Sports Exerc 2003 35 449 455 10.1249/01.MSS.0000053910.63105.45 12618575

[B12] Zawadzki KM Yaspelkis BBD Ivy JL Carbohydrate-protein complex increases the rate of muscle glycogen storage after exercise J Appl Physiol 1992 72 1854 1859 1601794 10.1152/jappl.1992.72.5.1854

[B13] Cadore EL Lhullier FL Brentano MA Da Silva EM Ambrosini MB Spinelli R Silva RF Kruel LF Hormonal responses to resistance exercise in long-term trained and untrained middle-aged men J Strength Cond Res 2008 22 1617 1624 10.1519/JSC.0b013e31817bd45d 18714223

[B14] Moore DR Del Bel NC Nizi KI Hartman JW Tang JE Armstrong D Phillips SM Resistance training reduces fasted- and fed-state leucine turnover and increases dietary nitrogen retention in previously untrained young men J Nutr 2007 137 985 991 17374665 10.1093/jn/137.4.985

[B15] Mori H Nakamoto M Kitagawa K Effect of simultaneous intake of protein and carbohydrate immediately after rugby training on body composition and various physical performance Eiyogaku Zasshi (Jpn Nutr Diet) 2010 68 173 182 (in Japanese) 10.5264/eiyogakuzashi.68.173

[B16] Mori H Differential effect of whey protein and carbohydrate intake timing after resistance training on nitrogen balance in trained young men Nippon Undo Seirigaku Gakkaishi (J Exercise Sports Physiol) 2013 20 55 64 (in Japanese)

[B17] Ministry of Health, Labour and Welfare Dietary Reference Intakes in Japanese 2010 Edition 2010 Tokyo: Ministry of Health, Labour and Welfare in Japanese

[B18] Kobayashi S Higuchi M Nutritional and Dietary Guidelines for Athletes 2006 Tokyo: Daiichi Shuppan Co. (in Japanese)

[B19] Tang JE Perco JG Moore DR Wilkinson SB Phillips SM Resistance training alters the response of fed state mixed muscle protein synthesis in young men Am J Physiol Regul Integr Comp Physiol 2008 294 172 178 10.1152/ajpregu.00636.2007 18032468

[B20] Rasmussen BB Tipton KD Miller SL Wolf SE Wolfe RR An oral essential amino acid-carbohydrate supplement enhances muscle protein anabolism after resistance exercise J Appl Physiol 2000 88 386 392 10658002 10.1152/jappl.2000.88.2.386

[B21] Boirie Y Dangin M Gachon P Slow and fast dietary proteins differently modulate postprandial protein accretion Proc Natl Acad Sci U S A 1997 94 14930 14935 10.1073/pnas.94.26.14930 9405716 PMC25140

[B22] Hoffman JR Ratamess NA Tranchina CP Rashti SL Kang J Faigenbaum AD Effect of protein-supplement timing on strength, power, and body-composition changes in resistance-trained men Int J Sport Nutr Exerc Metab 2009 19 172 185 19478342 10.1123/ijsnem.19.2.172

